# Development of a scoring system for non-specialist ratings of clinical competence in global mental health: a qualitative process evaluation of the Enhancing Assessment of Common Therapeutic Factors (ENACT) scale

**DOI:** 10.1017/gmh.2015.21

**Published:** 2015-12-09

**Authors:** B. A. Kohrt, M. K. Ramaiya, S. Rai, A. Bhardwaj, M. J. D Jordans

**Affiliations:** 1 Transcultural Psychosocial Organization (TPO) Nepal, Kathmandu, Nepal; 2 Department of Psychiatry and Behavioral Sciences, Duke University, Durham, USA; 3 Duke Global Health Institute, Duke University, Durham, USA; 4 Department of Psychology, University of Nevada, Reno, USA; 5 HealthNetTPO, Amsterdam, the Netherlands; 6 Centre for Global Mental Health, Institute of Psychiatry, Psychology, and Neuroscience, King's College London, London, UK

**Keywords:** Competence, culture, global health, measurement, task sharing, training

## Abstract

**Background.:**

Task-sharing is the involvement of non-specialist providers to deliver mental health services. A challenge for task-sharing programs is to achieve and maintain clinical competence of non-specialists, including primary care workers, paraprofessionals, and lay providers. We developed a tool for non-specialist peer ratings of common factors clinical competency to evaluate and optimize competence during training and supervision in global mental health task-sharing initiatives.

**Methods.:**

The 18-item **EN**hancing **A**ssessment of **C**ommon **T**herapeutic factors (ENACT) tool was pilot-tested with non-specialists participating in mental health Gap Action Programme trainings in Nepal. Qualitative process evaluation was used to document development of the peer rating scoring system. Qualitative data included interviews with trainers and raters as well as transcripts of pre- and post-training observed structured clinical evaluations.

**Results.:**

Five challenges for non-specialist peer ratings were identified through the process evaluation: (1) balance of training and supervision objectives with research objectives; (2) burden for peer raters due to number of scale items, number of response options, and use of behavioral counts; (3) capturing hierarchy of clinical skills; (4) objective *v.* subjective aspects of rating; and (5) social desirability when rating peers.

**Conclusion.:**

The process culminated in five recommendations based on the key findings for the development of scales to be used by non-specialists for peer ratings in low-resource settings. Further research is needed to determine the ability of ENACT to capture the relationship of clinical competence with client outcomes and to explore the relevance of these recommendations for non-specialist peer ratings in high-resource settings.

## Introduction

Task-sharing is the involvement of non-specialist providers in the delivery of mental health services that are, at present, predominantly performed by mental health professionals (Patel, 2009, 2012; WHO, 2008). In global mental health, non-specialist providers represent a broad category of professional and lay cadres who lack formal clinical training in fields such as psychology, psychiatry, and social work. Non-specialists may include social and community health workers, peer volunteers, teachers, midwives, traditional healers, and others lacking professional training experience. In low-and-middle-income countries (LMICs), examples of task-sharing include training community health workers and persons without a professional health role to diagnose and manage common mental disorders and deliver specialized psychological treatments including interpersonal therapy, behavioral activation, and cognitive processing therapy (van Ginneken *et al.* 2013).

Lack of properly trained personnel is a key barrier to dissemination, implementation, and scaling up of task-sharing programs (Murray *et al.* 2014*a*). The limited number of mental health specialists presents a challenge not only for evidence-based delivery of mental health care but also for training and supervision. Clinical resources for training and supervision—both in terms of human capacity and materials—are historically lacking in most LMIC settings (Abas *et al.* 2003; Kakuma *et al.* 2011). Therefore, there is a need for task-share quality improvement activities with non-specialist providers as well.

One means of addressing this barrier to scaling up services in global mental health is utilizing culturally adapted tools to evaluate therapist competence. Therapist competence reflects how well a health worker implements a technique and implies both adhering to an evidence-based treatment and implementing it with appropriate skill and is typically measured with structured role plays using standardize patients; therapy quality refers to how well a treatment is delivered in a clinical setting with real patients (Margison *et al.* 2000; Fairburn & Cooper, 2011). Without easily administered assessments of non-specialist competence, it is challenging to determine both the minimum skill level necessary for effective delivery of evidence-based treatments, as well as the success of training and supervision practices on the development and maintenance of these competencies. One innovative approach is the use of instruments to assess therapist competence that can be administered by non-specialist peers to promote quality improvement (Singla *et al.* 2014), rather than using tools requiring trained experts for administration and scoring.

In high-income settings, there are a wide variety of instruments available for expert ratings of therapeutic competence and quality (Margison *et al.* 2000; Cahill *et al.* 2008). These vary in length, with some tools containing 12 items, e.g. Cognitive Therapy Scale-Revised, CTS-R (Blackburn *et al.* 2001), and others containing up to 70, e.g. Hill Interaction Matrix-Group, HIM-G (Hill & Gormally, 1977), or more, e.g. Vanderbilt Therapeutic Alliance Scale (O'Malley *et al.* 1983), (see Table 1). Response options are typically Likert scales that reflect the degree of agreement (e.g. ‘strongly disagree’ to ‘strongly agree’) with a declarative statement. Other tools include frequency markers or behavior counts. Response alternatives vary from five-point scales, e.g. Cross-Cultural Counseling Inventory-Revised, CCCI-R (Lafromboise *et al.* 1991), to nine-point scales, e.g. Therapist post-session Questionnaire (Samstag *et al.* 1998), with most containing seven response options.

**Table 1.** Examples of response options for therapist rating scales

Tool	Number of items	Response options
Coding the Interaction in Psychotherapy, CIP (Schindler, Hohenberger-Sieber, & Hahlweg, 1989)	19 items	Relative frequency response with behavior counts
CTS-R (Blackburn *et al.* 2001)	12 items	0–6 scale, with skill-based descriptions for each response presented in hierarchy of mastery
CRF-S (Corrigan & Schmidt, 1983)	12 items	7-point scale with blank spaces for ‘X’ marking, with one anchor point at either end for ‘not very’ and ‘very’
CCCI-R (Lafromboise *et al.* 1991)	18 items	5-point Likert scale, ‘strongly disagree’ to ‘strongly agree’
Helping Alliance Questionnaire revised, HAq-II (Luborsky *et al.* 1996)	19 items	1–6 Likert scale, ‘strongly disagree’ to ‘strongly agree’ with anchor points for each score
HIM-G (Hill & Gormally, 1977)	72 items	0–5 scale, with various response sets; e.g. frequency ‘not at all’, ‘0–10% of the time’, … ‘over 60% of the time’; number of people ‘most people,’ ‘few people’, … ‘nobody’
Jefferson Empathy Scale, JES (Suh, Hong, Lee, Gonnella, & Hojat, 2012)	20 items	7-point Likert scale, ‘strongly disagree’ to ‘strongly agree’
Maslach Burnout Inventory, MBI (Maslach, Jackson, & Leiter, 1996)	22 items	0–6 scale for frequency, ‘0 = never’, ‘6 = everyday’
Rater Applied Performance Scale, RAPS (Lipsitz *et al.* 2004)	6 items	5-point scale, ‘not applicable’, ‘unsatisfactory’, ‘fair’, ‘good’, ‘excellent’
Therapist Action Tool, TAT (Hoyt, Marmar, Horowitz, & Alvarez, 1981)	25 items	0–5 scale with four anchor points, ‘0 = did not do it’, ‘1 = occurred but minor’, ‘3 = moderate’, ‘5 = major emphasis’
Therapist Post-Session Questionnaire (Samstag *et al.* 1998)	40 items	A variety of response sets, e.g. 1–9 Likert scale, categorical responses, open-ended, and 7-point dyadic attributes (bad to good, safe to dangerous)
Vanderbilt Therapeutic Strategies Scale, VTSS (Henry *et al.* 1993)	21 items	5-point scale with frequency responses for some items and degree of quality responses for other items

Our goal was to develop a novel rating scale that would be appropriate for peer ratings by non-specialists in a global mental health context. Despite the abundant existence of peer rating scales in high-income settings, there are a number of concerns that these instruments may have limited applicability and relevance in LMICs. For instance, Likert scale peer rating instruments capture subjective feelings relative to a non-representative comparison group, thereby limiting their ability to capture absolute or context-free judgment at the population level (Biernat *et al.* 1991). Research in cross-cultural psychology echoes these concerns regarding Likert scales (Heine *et al.* 2002). Further, instruments containing too many response alternatives are associated with increased cognitive load, fatigue, and response bias (Anastasi, 1976; Krosnick, 1991), as well a decrease in ability to accurately discriminate between options (Miller, 1956). These concerns may be magnified for non-specialists with limited-to-no prior training in psychological instrument administration.

Dissemination and implementation theory calls for tools to be context-appropriate (Murray *et al.* 2014*a*), thus requiring task-sharing instruments in LMICs to be consistent with the objectives and resources of the prevailing cultural group. Across studies and interventions ‘common factors’ refer to those processes, which contribute to positive patient outcomes regardless of specific treatment features. Although definitions vary (Frank & Frank, 1991; Wampold, 2011), common factors often refer to therapist qualities and therapist–client interactions including empathy and genuineness, client and extra-contextual factors such as mobilization of social support and bolstering of prior positive coping strategies, and aspects of the therapist–client relationship including collaborative goal setting and overall promotion of hope and expectancy of change among clients (Garfield, 1973; Greenberg, 2004; Sparks *et al.* 2008; Karson & Fox, 2010; Wampold, 2011).

Despite this importance of common factors for positive patient outcomes, measurement of therapist competence related to common factors in LMICs has been limited, with few researchers systematically adapting or developing novel tools for use by non-specialists (Kabura *et al.* 2005). Working with non-specialists in Uganda, Kabura *et al.* (2005) adapted the Attending Behavior Rating Scale (Ivey & Authier, 1978), which is used to rate micro-counseling skills: eye contact, vocal tone, posture, and verbal attending behavior. The Uganda adaptations included adding specific frequency counts for behaviors such as open-ended questions, paraphrasing, and reflecting feelings. However, no cultural adaptations were described. Studies using World Health Organization (WHO) recommendations for mental health integration in primary care have included observed evaluations of therapy quality in actual clinical practice, but role plays with standardized patients for competence evaluation were not reported (Sadik *et al.* 2011; Makanjuola *et al.* 2012; Jenkins *et al.* 2013). Overall, there has been a lack of adaptations and novel tool development for non-specialists to evaluate competence in cross-cultural settings.

To address this gap in availability of tools for global mental health, we have developed a tool to use during training and supervision in LMICs to assess common factors competence among non-specialist providers delivering mental health services (Kohrt *et al.* 2015). This tool, the **EN**hancing **A**ssessment of **C**ommon **T**herapeutic factors (ENACT) rating scale, was developed using a four-step systematic process in Nepal. We have previously described the systematic process of item generation, piloting, and basic psychometric properties (Kohrt *et al.* 2015).

### Objective

In this paper, we focus on the development of the scoring system for ENACT, with the goal of describing the process to optimize feasibility and utility among non-specialist peer raters in a LMIC cultural context. We present a qualitative process evaluation that led to a three-tiered competency rating system designed for use in task-sharing initiatives in global mental health. The findings are relevant to guide development of non-specialist peer ratings for global mental health in low-resource settings.

## Methods

### Tool development

ENACT was developed in the context of the Program to Improve Mental Health Care (PRIME), an initiative in LMICs to develop mental health services in primary care and community settings (Lund *et al.* 2012; Jordans *et al.* 2013). In Nepal's Chitwan District, primary care and community health workers are being trained with a locally developed Mental Health Care Package (Jordans *et al.* 2015), which includes WHO's mental health Gap Action Programme (*mhGAP*) *Intervention Guide* (*mhGAP-IG*) (WHO, 2010), psychosocial skills modules, and brief modified versions of behavioral activation (the Healthy Activity Program) and motivational interviewing (Counseling for Alcohol Program) (Patel *et al.* 2014; Singla *et al.* 2014). The target settings in Nepal include health posts, sub-health posts, and primary health centers. These are the first port of entry for the general public seeking medical care. All trainees in PRIME are non-specialists working in these settings. The trainers for primary care workers include Nepali psychiatrists trained on mhGAP-IG and Nepali psychosocial counselors with a decade of experience in mental health programs and more than 5 years of clinical supervision work.

ENACT (see full tool in online Supplemental Material) was developed using a four-step process within PRIME (Kohrt *et al.* 2015). First, a review of 56 client–therapist interaction instruments was used to generate a pool of common factors. Role-plays between Nepali therapists and standardized clients were used to generate additional domains of cultural and clinical significance. Then 10 Nepali therapists scored the initial domains for comprehensibility and clinical significance. Domains with high scores in both of comprehensibility and clinical significance were pilot tested with two expert Nepali therapists using brief standardized role-plays. Session transcripts were then qualitatively coded to examine the instrument's feasibility, acceptability, and reliability. After further domain consolidation and revision, a list of 18 unique items was generated. The final 18-item tool incorporated a 3-tiered response system 1 (‘needs improvement’), 2 (‘done partially’), and 3 (‘done well’). Psychometric measurements of the 18-item instrument followed. The intra-class correlation coefficient (ICC) for expert therapists was established through rating videotaped sessions: ICC (2,7) = 0.88 (95% CI 0.81–0.93). Non-specialist peer ICC (1,3) based on post-training role-plays was 0.67 (95% CI 0.60–0.73). Cronbach's alpha based on 34 expert ratings of non-specialist roles plays was 0.89. Cronbach's alpha for non-specialist peer-ratings was 0.80 (*N* = 113).

### Process evaluation

**Fig. 1.**
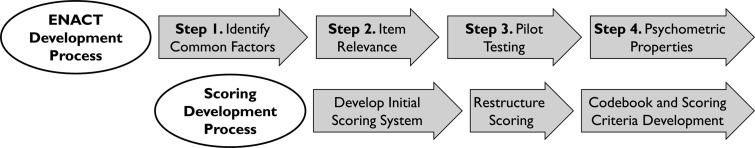
Scoring guideline development process within the ENACT tool development process.

In this paper, we present the qualitative process evaluation that led to development of a three-tiered response structure designed to optimize feasibility and utility for peer raters. We describe the challenges encountered during scoring system development and how these were addressed. Scoring guidelines were created in an iterative process conducted in tandem with the overall ENACT protocol (See Figure 1).

### Development of an initial scoring system

**Fig. 2.**
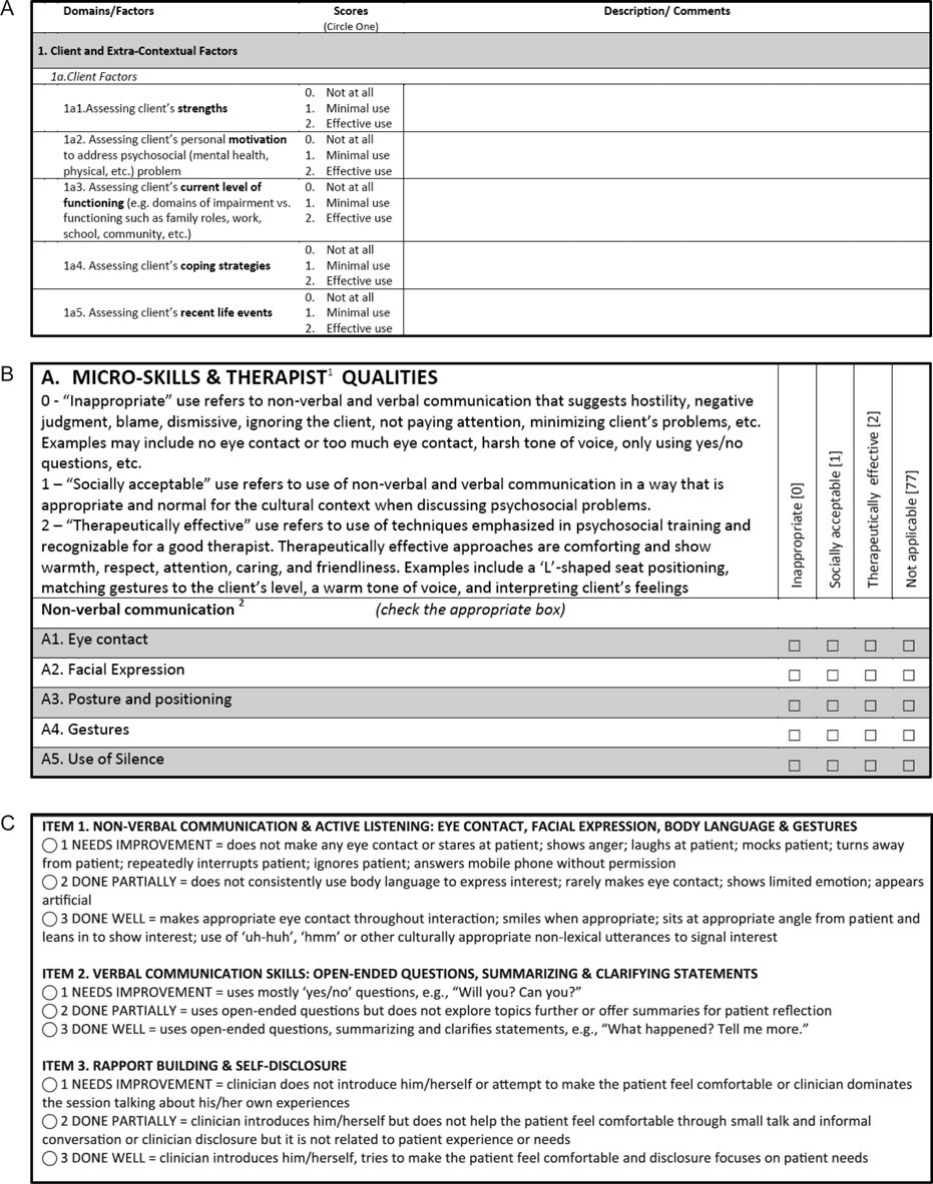
First (A), second (B), and final (C) iterations of scoring system for ENACT.

After Step 1 (domain generation) and Step 2 (domain relevance) of ENACT development, a 49-item tool was created for piloting. A three-tiered scoring system containing the values 0 ‘not at all’, 1 ‘minimal use’, and 2 ‘effective use’ was selected for rating (see Figure 2). We initially chose a three-point scale as the minimally sufficient response option set. If the three-point scale had high feasibility and utility in proceeding steps, we would conserve it. If, however, it were deemed inadequate, additional points could be added to achieve the minimum number of response options required to capture variance and change in competence throughout the training and supervision process. This strategy was chosen based on prior literature suggesting cognitive burden and discriminatory impairments associated with an increase in response options (Miller, 1956; Anastasi, 1976; Krosnick, 1991). We viewed these concerns as particularly salient among non-specialist raters. We also chose to use qualifiers of skill performance (minimal use, effective use) over Likert ratings of agreement based on prior cross-cultural research on the limitations of the latter (Heine *et al.* 2002). Our prior work adapting psychiatric symptom scales in Nepal also highlighted problems with the use of Likert scales due to a cultural preference for agreement and acquiescence over disagreement ratings (Kohrt *et al.* 2011).

### Pilot testing and scoring system revision

Government primary health workers who received mental health trainings participated in observed structured clinical evaluations (OSCEs), with standardized role-plays before and after the PRIME training. All role-plays in the study ranged from 15–20 min and covered a range of common clinical presentations including depression and self-harm. Role-play vignettes were developed by the study team (two Nepali therapists, Nepali study coordinator, Nepali training supervisor, and expatriate researchers—a psychiatrist and a psychologist—with greater than 10 years of experience working in Nepal). The vignettes were based on Nepali therapists’ experiences with actual client interactions, for further information on vignette development, see (Kohrt *et al.* 2015). Vignettes were designed so that all domains on the ENACT scale would be applicable. The vignettes were then used to train Nepali psychosocial therapists to play the part of a standardized client.

OSCE role-plays were audio taped for transcription, translation, and qualitative data analysis (described below). After each rating, focus group discussions (FGDs) were conducted to qualitatively explore the feasibility, utility, and reliability of the scoring guidelines. FGDs included trainers, research coordinators, research assistants involved in trainings, the principal investigator, and a consultant. Process notes were recorded for analysis and to guide adaptation of the tool. The result of this process was a revision of the initial scoring system. Over multiple iterations, the tool was reduced from 49 to 18 items and the scoring system was revised.

### Development of scoring criteria and an ENACT codebook

During the piloting phase, scoring criteria were modified and a preliminary codebook was developed. After finalizing the 18-item scale, an accompanying codebook was created. Data sources included observation of 30 primary care trainees enrolled in trainings, 26 standardized patient role-plays with non-specialists, and four expert sessions with actual clients (see Table 2). Of the standardized role-plays, 19 were post-training and seven were pre-training. All of the actual client sessions were conducted with an expert therapist who had more than 5 years of clinical experience. The audio recordings were transcribed in Nepali and translated into English. We employed a content analysis approach for the coding. In our prior review of common factors in existing instruments combined with common factor domain generation with Nepali counselors, we identified 115 common factor domains (Kohrt *et al*. 2015). These domains were used for the content analysis of the transcripts. This represented a deductive coding process because the individual codes had been established previously from common factors literature.

**Table 2.** Non-specialist trainees completing pre- and post-training observed structured clinical evaluations with standardized clients

Non-Specialist (NS)#	Age	Gender	Level of health training
NS #01	40	Male	Community medical auxiliary
NS #02	39	Male	Health assistant
NS #03	40	Male	Community medical auxiliary
NS #04	51	Male	Health assistant
NS #05	44	Male	Health assistant
NS #06	26	Male	Health assistant
NS #07	51	Male	Community medical auxiliary
NS #08	40	Male	Community medical auxiliary
NS #09	38	Male	Community medical auxiliary
NS #10	38	Male	Community medical auxiliary
NS #11	40	Male	Community medical auxiliary
NS #12	38	Female	Auxiliary health worker
NS #13	35	Female	Community medical auxiliary
NS #14	40	Male	Community medical auxiliary
NS #15	40	Male	Community medical auxiliary
NS #16	43	Male	Community medical auxiliary
NS #17	53	Male	Community medical auxiliary
NS #18	50	Male	Community medical auxiliary
NS #19	25	Female	Community medical auxiliary
NS #20	41	Male	Community medical auxiliary
NS #21	50	Male	Community medical auxiliary
NS #22	29	Female	Community medical auxiliary
NS #23	45	Male	Community medical auxiliary
NS #24	30	Female	Community medical auxiliary
NS #25	34	Male	Community medical auxiliary
NS #26	38	Male	Community medical auxiliary
NS #27	21	Male	Community medical auxiliary
NS #28	39	Male	Community medical auxiliary
NS #29	38	Male	Community medical auxiliary
NS #30	51	Male	Community medical auxiliary

The qualitative data analysis software NVivo was used for all coding (QSR International, 2012). English transcripts were triple coded in NVivo by three study authors including one American psychiatrist, one Nepali psychosocial researcher, and one American graduate student. After collective development and revision of the codebook, transcripts were rated independently. Raters were not blinded to pre- *v.* post-training status of the transcript. Interrater reliability greater than 0.80 was required for each domain among the three coders.

Coding results were used to develop scoring criteria for each of the 18 items on the ENACT scale. Because this research was conducted within a task-shifting framework, criteria were particularly detailed so that they were easily comprehensible by non-specialists in similar lower-and-middle-income settings. Following criteria development, we consolidated results into an ENACT codebook for use by future expert raters. Codebook contents included detailed scoring criteria, along with culturally grounded examples of each of the 18 domains that exemplified a specific scoring level. Rationales for each example were provided. Below, we present the challenges encountered during development of the scoring system as documented through the process notes and standardized client role-plays.

## Results

### Trainees’ perception of tool objectives: training and supervision *v.* research

One of the first challenges in implementing ENACT was the motivation of non-specialist providers to take part in standardized role-plays and conduct peer ratings. Without motivation to participate in role-plays, and complete the full version of the tool, the instrument would not contribute to quality improvement among peers. During the first application of the tool, one trainee, a health officer in charge of a primary care setting, initially refused to use the tool. This led us to revise how we introduced and explained ENACT in future trainings. We emphasized the tool's role in quality improvement for training and supervision to counter perceptions that it was only an instrument intended for research. For example, we changed our description of the tool by including relevant slides and examples of how it directly interfaced with common factors and psychosocial skills taught during the training. Another major change was to redesign the tool's response options, which were limited to the vague statements ‘not at all’, ‘minimal use’ and ‘effective use’. After the third iteration, there were full descriptions of the three tiers of performance for each item (see Figure 2). Through this approach, the tool appeared more as a teaching tool that reinforced the ideal level of skill required to achieve mastery in each domain.

An example of using the tool to reinforce competency goals can be seen through ENACT item #9 ‘assessing recent life events and impact on psychosocial wellbeing’. Tier 1 was characterized by sessions in which the provider ‘does not ask about triggering life events.’ For a score of 2, the provider ‘asks about life events but does not connect with current mental health needs’. For the highest score the provider ‘asks about life events and discusses connection with current mental health needs’. By including a finer grain level of detail, peer raters could immediately see specific requirements for ‘done well’. In prior scoring descriptions such as ‘inappropriate’ *v*. ‘therapeutic’, peers were not able to recall what the ideal skill application should be. The transcript below illustrates a provider who helps the client identify potential causes for insomnia and its relation to a buffalo loss, thus earning a score of 3 (of note, although scoring was assigned for each domain upon reviewing an entire session, the result below and subsequent quotes provide only a snapshot of how particular aspects of a domain were categorized into three competency tiers).

Client: I have been having problems sleeping for a long time. I don't enjoy working and have lost my appetite. It has been around eighteen days.

Provider: It has already been eighteen days. What could be the reason for this?

Client: I don't know.

Provider: Why do you think it is hard to sleep?

Client: Hmm, what are the reasons for that?

Provider: Do you have any problems at home?

Client: Yes, I do.

Provider: And what are they?

Client: I had a buffalo, which gave me lot of milk. It fell down and died.

Provider: How long has it been?

Client: It has been around twenty-five to thirty days. It died. I had invested around forty thousand (US$400) on it. Because of this, my life is sorrow and hardship.

[NS #03, 40-year-old male community medical auxiliary, post-training role-play]

### Tool length

Trainers reported that completing the initial 49-item ENACT version was time-consuming for both trainers and trainees due to the high number of items. Further, they argued that it was difficult to keep all of the items in mind when observing structured clinical evaluations with standardized clients. Subsequently, one approach to item reduction was to employ the qualitative coding to group items together by clustering skills. The qualitative coders identified such clusters. For example, the item ‘collaborative goal setting’ and ‘addressing client expectations’, when done well were typically done together.

Therefore, for ENACT item #12, we categorized sessions in which the clinician dictated treatment goals and plans without client consultation as 1. For the second-tier, the provider asks about client goals, but does not discuss their feasibility or implementation. For the highest score, the provider and client jointly identify goals, discuss feasibility, and make action plans. Here, the provider works with the client to develop treatment goals and then to select a strategy for implementation. The provider highlights the client's agency in modifying goals and strategies (e.g. by increasing frequency of sessions), thus earning a score of 3.

Provider: So right now you are having a misunderstanding with your wife and have started drinking.

Client: Yes. I don't want to go back home early as I do not want to argue with my wife and so I always end up getting drunk at my friend's place. I have started drinking a lot.

Provider: So which issue do you think is important to address first?

Client: I think if I have a good relationship with my wife, I can cut down my drinking.

Provider: Okay, so then we will work on improving your marriage. We will meet every week, develop our plan of action and then review it and develop another plan in the next session. What do you think?

Client: I think that is good. But, I think it would be better if we could meet sooner: maybe twice a week?

Provider: Sure, we will meet every four days then.

[NS #05, 44-year-old male health assistant, post-training role-play]

### Hierarchy of skills

One of the modifications to the tool that incorporated both the need to collapse items and the need to have the tool serve an educational function was to create a hierarchy of clinical skills within the scoring system. In the tool's initial version, there were 49 individual items with the associated scoring responses ‘not at all’, ‘minimal use’, and ‘effective use’. Through qualitative coding, we found that numerous skills occurred together in a progression, where demonstration of higher order skill or a sequential skill routinely occurred after a lower order skill was displayed. For instance, there were initially separate items referring to exploration, interpretation, and normalization of feelings. However, interpretation and normalization occurred only if feelings were explored. Therefore, for ENACT item #4, we grouped these items together into one item within a three-tiered hierarchy, and we were able to capture emotionally judgmental and critical provider behaviors as well.

For ENACT item #4, a score of 1 refers to not asking about feelings or making judgmental, critical, or dismissive statements about emotions. A score of 2 categorizes statements where emotions were elicited but not normalized. A score of 3 refers to appropriate eliciting, exploration, and normalization of the client's emotional experience. In the example below, the provider helps the client explore and interpret her feelings and their associated contexts. The provider normalizes the happiness the client feels in her ‘heart-mind’ while present with her child compared with other activities. Heart-mind refers to the Nepali center of emotion, memory, and individuality (Kohrt & Harper, 2008). This combination illustrated below would be consistent with a score of 3.

Provider: May I know about your daily routine?

Client: Yesterday morning, I woke up at around six. Then I washed my face. I didn't even eat anything.

Provider: When you woke up, did you feel that your head was heavy? Were you sad or happy then?

Client: My head was very heavy.

Provider: After you washed your face, did you feel happy and fresh?

Client: I didn't notice that.

Provider: So continue, what did you do during the daytime?

Client: I washed my face and drank tea. I have a child. My child woke up and I prepared to feed him milk. My heart-mind was happy then.

Provider: So your heart-mind was happy when you were taking care of your child? What did you do after that?

Client: After that, I came to the office at eight in the morning. I am at the office from eight to five. I forget these things while I am working.

Provider: While you were busy in your office, did you feel happy or sad?

Client: I don't remember, as I was too busy in office work. When I returned back, I enjoyed being with my child. After that I tried to sleep but couldn't at all.

Provider: Okay, you say this is your problem. You did not get sleep. You feel happy when you are playing with your child, right?

Client: A lot.

Provider: Being mothers, we feel happy playing with our children. You became happy while you were playing with your child. Can you tell me anything besides that when you became happy yesterday?

[NS #12, 38-year-old female auxiliary health worker, post-training role-play]

Similarly, we were able to group four items on collaborative problem identification and solving into one item (ENACT item #15) with the three tiers reflecting sequential completion of steps required for effective problem solving.

### Subjective *v.* objective ratings

Because of concerns regarding rating subjectivity, the first incarnation of the ENACT scale did not include Likert agreement responses. In prior work in Nepal, we found that the concept of agreement (e.g. totally agree *v.* somewhat agree) was seen as a personal judgment rather than an objective observation and therefore would interfere with making and sharing peer ratings. In addition, during piloting we found that both expert trainers and peer trainees had questions regarding objectively rating concepts such as ‘promoting realistic hope for change’ (ENACT item 13). Therefore, detailed scoring descriptions were required. The lowest tier included interactions where the provider does nothing to establish hope for change or, in the Nepali context, establishes unrealistic goals for improvement (e.g. telling a client with chronic psychosis that he/she will undoubtedly be cured in 3 months and can then stop medication forever). The second-tier score refers to referencing vague, positive outcomes, and promoting unrealistic hope. The highest tier is the development of hope that is reasonable, exemplified by discussing specific, context-appropriate treatment goals.

The account below includes an assessment of whether or not the client feels she can get better. However, the provider then makes a generalizing comment that fosters unrealistic expectations for change. Therefore, this account would be 2.

Provider: Do you think this is a situation that can get better, or do you believe it will always be this way?

Client: I think this is just how life is and will be. I've been sad for a long time. I don't believe things can change.

Provider: Well, you showed up for therapy today. Don't you think that means you might have some hope? If we are doing the right thing then what is there to be afraid of? I don't think your friends won't understand. Your friends are literate, have social status, and you are also in that group. If you don't want to stay away from your friends, then you can bring your friends too and we will discuss. You can tell them that if they too stay away from drinking then things will be better. It will be easier. Now we have to first make a plan. You have come here with a deep desire of quitting drinking. This is a plus point. Let us now make a plan and accordingly you can work on decreasing drinking. Then we can be successful. When can we meet again? Let's meet again and discuss so that I can help in making your effort successful.

Client: I will come after a week. You talked about my friends. No matter what, they are still friends. I will try to talk with them once. Let's see what they say?

Provider: So you will come after a week. Try to decrease your drinking level too. Try to control your heart-mind. Okay?

Client: I will try.

Provider: If you try, you will succeed. There is nothing that you cannot do.

[NS #20, 41-year-old male community medical auxiliary, post-training role-play]

### Behavior counts

In attempt to promote objectivity, we attempted to use behavior counts for the different scoring levels. If a behavior was done ‘X’ number of times, the score was 2 ‘done partially’, *v.* ‘Y’ number of times resulting in a score of 3 ‘done well’. For example, for ENACT item 2 ‘verbal communication skills’, the qualitative coders characterized Tier 1 ratings as those including fewer than two open-ended questions between provider and client, no summarizing statements, or no attempt at clarification with the client. Meeting any of these criteria led to a score of 1. Tier 2, was characterized by use of two or more open-ended questions, at least one summarizing statement, and at least one clarification statement. Tier 3 included meeting Tier 2 criteria plus greater than two summarizing statements. We chose to include these behavior counts because a similar approach was used with Uganda paraprofessional counselor evaluation (Kabura *et al.* 2005).

However, we found that both expert raters and participants had difficulty keeping exact behavioral counts in mind. Therefore, the response options were changed to reflect increasingly sophisticated communication skills moving from closed-ended questions (Tier 1) to open-ended questions (Tier 2) to both open-ended questions and summarizing statements (Tier 3), but without reference to the exact number of such occurrences.

The selection below is from a health worker trainee in a standardized role-play. The selection is taken from a role-play that scored 3 based on repeated use of summarizing, open-ended questions, and clarifying statements.

Provider: I will go back in time. How long has it been since that you got married?

Client: It has been around 15–16 years.

Provider: Oh, it has already been 15–16 years. That means you have two children, a husband, and have been married for 16 years. Can you tell me how your relationship is with your husband? Your relationship with your husband is now nearly 2 decades long. But still, if you can say something about your relationship with your husband.

Client: It is not that good. It is just okay.

Provider: You said it is not that good. Can you explain how it is?

Client: He does not listen to me nor take my advice. He dominates me a lot.

Provider: That means even though you have been together for 16 years after marriage, you don't share a lot with your husband. Because of that, you are feeling that your husband is not helping and loving you. How is your husband's relationship with people outside your home? Do you have any doubts?

[NS #24, 30-year-old female community medical auxiliary, post-training role-play]

### Use of ‘not applicable’ rating option

In the initial scoring system design, ‘not applicable’ was included as a response option. However, we found that this response was often selected in error. For example, when trainees failed to rate item #18 ‘safety and suicidality’, peers would rate the item as not applicable rather than scoring as ‘needs improvement’, the lowest tier. Moreover, a significant amount of time in trainings and scoring was also lost due to debates about what was considered ‘not applicable’. Therefore, we created role-plays in which all 18 items of the ENACT were applicable. Trainees were then instructed to place a score of ‘needs improvement’ if the action was not done.

### Social desirability and response set options

From the outset, three response options were used because of our intention to include the fewest number of response options required to capture adequate variance and change over time. In the first iteration of the tool, the three response options were 0 ‘not at all’, 1 ‘minimal use’, 2 ‘effective use’. This structure became difficult to employ because it only was appropriate for items where we were focusing on the presence of a behavior. It, however, was not meaningful when one intended to capture behaviors that should be avoided. Therefore, in the second incarnation, the scoring was 0 ‘inappropriate’, 1 ‘socially acceptable’, 2 ‘therapeutically effective’, and 77 ‘not applicable’. However, participants reported that this approach was too subjective. Based on caste/ethnicity, age, gender, occupation, and other demographic markers, what is considered ‘socially acceptable’ and ‘inappropriate’ may vary. In the third format, we employed 0 needs improvement, 1 done partially, and 2 done well. Although the wording was considered acceptable, we initially found very few 0 responses. When non-specialists were asked about this, they reported that it was unacceptable to rate their peers as 0. We then maintained the text descriptions but changed the numbers to 1–2–3 instead of 0-1-2. By changing 0–1 and shifting the scale upwards, we found an increased use of the lowest score option 1.

## Discussion

The qualitative process evaluation during development of the scoring system for ENACT revealed challenges in the creation of peer rating tools for non-specialist providers. The challenges can be grouped into five domains: (1) balance of training and supervision objectives with research objectives; (2) burden for peer raters due to number of scale items, number of response options, and use of behavioral counts; (3) incorporating a hierarchy of clinical skills; (4) objective *v.* subjective aspects of rating; and, (5) social desirability when rating peers.

### Five challenges for non-specialist peer rating tools

**Fig. 3.**
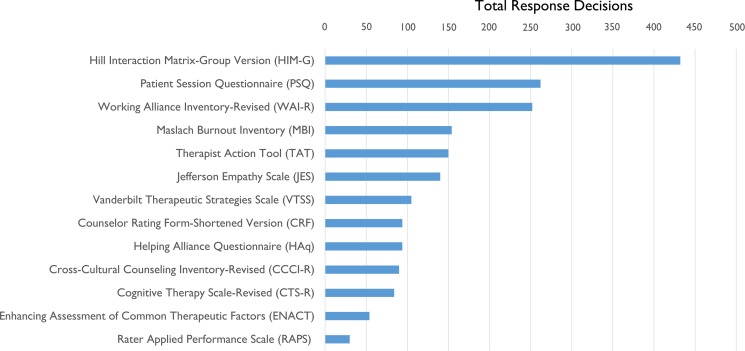
Total response decisions for therapist rating tools. Total response decisions refer to number of items multiplied by the number of response options per item.

(1) *To facilitate adoption of tools for quality improve purposes, the value of tools for training and supervision needs to be clarified beyond use as research instruments*. ENACT needed to represent a teaching aid. This led to changes regarding details for each item specifying what was meant by ‘done well’ *v*. ‘needs improvement’. The CTS-R is a one example of this in the current literature, as it provides information and key features of each skill and descriptions for each scoring level (Blackburn *et al.* 2001). Tools such as Counselor Rating Form-Shortened version, CRF-S (Corrigan & Schmidt, 1983) that use general responses such as ‘very’ to ‘not very’ may have less appeal for peer non-specialist ratings because the direct relevance to clinical improvement and the educational value of the tool is more opaque.
(2) *Regarding scalability, tools need to be user-friendly, which includes limiting the number of items and the number of response set options, as well as avoiding taxing concentration with a range of behavioral counts*. Tools with nine Likert response options or 100 items are less amenable to non-specialist peers who are burdened with a range of other healthcare or social work duties. In Figure 3, we compare ENACT with other therapist rating tools based on the total number of scoring decisions (total number of items X number of response options per item) as approximation of the burden on cognition and time. At the extreme upper end is the HIM-G version with over 400 response decisions, exemplifying a tool that gathers a tremendous amount of data but is limited to use by experts. The Patient Session Questionnaire and Working Alliance Inventory-Revised have over 200 response decisions, thus also burdensome for peer non-specialists. A number of existing tools have approximately 90 response decisions (CRF-S version, Helping Alliance Questionnaire, CTS-R). ENACT was designed to have a minimal burden on peers with 54 response decisions (18 items with three response options per item). Despite successful use of behavior counts in a Ugandan study (Kabura *et al.* 2005), participants in Nepal reported difficulty keeping track of counts when making live ratings. We also dropped ‘not applicable’ because the time and confusion related to when it should be invoked.
(3) *One component of optimizing training and supervision utility as well as minimizing the number of items is to use a scoring system that captures skills hierarchies*. The CTS-R is one of the few tools that incorporates this. Other tools using Likert agreement endorsements per items do not distinguish between degree to which a simple skill was done well and the level of complexity with which that skill was demonstrated.
(4) *When rating peers and working with non-specialists, it was important to minimize perception that scoring represents subjective personal appraisals or opinions*. Likert agreement scales were not selected and detailed descriptions were needed so that peers could point toward specific behaviors that justified a score. Moreover, by anchoring each score with a description, it alleviates anxiety and time burden related to distinguish between scores such as a ‘5’ and ‘6’ on a nine-item Likert scale that has a limited number of anchors. This may be especially important in cultural context where group consensus is emphasized (Heine *et al.* 2002).
(5) *When asking peers to rate one another, social desirability biases need to be considered.* Through small changes such as using 1 as the lowest score with a description of ‘needs improvement’, we were able to increase use of this response option compared with when the lowest score was 0 described as ‘not at all’ or ‘inappropriate’.

Taken together, these issues engage with global mental health debates regarding the tension between ‘excellence’ and ‘relevance’, with researchers focusing on detailed, elaborate information, and practitioners interested in issues of application and ‘good enough’ services (Tol *et al.* 2012). ENACT was developed to provide information to contribute to excellence in research, but with a scope and burden that would enable it to be relevant for non-specialists to put into practice, thus increasing the likelihood of scalability.

### Application of ENACT scoring system

ENACT's scoring system lends itself to a number of applications. The scoring system can be used to guide decision-making. Tier 1 reflected the need for significant improvement and remediation through training and supervision. For situations where minimum competence is required, e.g. participating in a research trial, then persons achieving only or mostly Tier 1 competency (post training) across many domains may not be selected. Tier 2 demonstrates partial competence of a common factor. Program designers could anticipate Tier 2 competencies to be achieved in most domains after training has been completed but before a protracted period of supervision begins. Following supervision, Tier 3 would be a reasonable expectation. Tier 3 is the optimal level for basic competence in a common factor. After training and supervision for a pre-determined period (e.g. 3–6 months), non-specialists would be expected to achieve tier 3 on most domains.

In addition, ENACT can be used to identify specific competencies which could be considered absolute minimal requirements for involvement in task-sharing care delivery. For example, Murray *et al.* (2014*b*) identified safety planning for risk of harm (self-harm, intimate partner violence, and child maltreatment) as key domains in task-sharing which are often neglected. Therefore, third-tier competency on ENACT item #18 could be a minimum requirement for practice. Similarly, certain task-sharing programs may emphasize specific common factors more than others do based on their theory of change models and implementation. For example, the ‘Friendship Bench’ program in Zimbabwe relies strongly upon problem solving (Chibanda *et al.* 2011), and thus, achievement of third-tier competency would be a minimum requirement on ENACT item #15.

From an implementation science and program evaluation perspective, the percentage of non-specialists achieving competency on each item can be used to highlight areas for change. For example, if there are certain items where most non-specialists achieve only Tier 1 following training, training content would need to be improved in that domain. If, after 6 months of supervision most non-specialists were still at Tier 2, then supervision intensity and strategy should be adjusted. Tools such as ENACT can be used to enhance apprentice-based supervision strategies that focus on individual provider needs and skill development (Murray *et al.* 2011); for example, ENACT can be used to identify which common factors are at the lower competency tiers and select them for structured remediation.

The applications of ENACT are not limited to LMICs. ENACT and similar endeavors are important for high-income countries (HIC), such as countries in North America and Europe, where there is also significant burden of unaddressed mental health needs. Gaps in services are especially pronounced among racial and ethnic minority groups (Alegría *et al.* 2008). Tools built for cross-cultural use could be ideal for non-specialists from these communities delivering mental health services. For many populations in HIC especially minority ethnic groups, primary care and community-based providers are more likely to be delivering mental health services (Wang *et al.* 2006). These providers may be preferred because of financial barriers, lack of transportation, stigma associated with mental health professionals, and other cultural issues in explanatory models and help-seeking behavior that limit use of specialty mental health services (Scheppers *et al.* 2006).

For non-mental health specialists in HIC to adopt quality improvement tools for mental health, tools need to address the five challenges identified in our results presented here. Based on our review of existing 56 common factors tools developed in high-income settings (Kohrt *et al.* 2015), the majority of tools do not adequately counter these potential barriers. Therefore, ENACT and similarly developed tools should be explored for use with non-specialist providers in high-income settings. Tools such as ENACT can be useful to evaluate competence among primary care workers, community health workers, peer helpers, cultural brokers, refugee resettlement workers, teachers, and paraprofessionals. This raises an important distinction between tools to assess cultural competence of expert therapist (of which there are many examples) and tools that are cross-culturally appropriate for rating common factors among diverse care providers with limited mental health expertise. ENACT fills a gap in this latter arena.

Moreover, because of the debates regarding use of non-specialists in HIC focuses on concerns about quality of care (Robiner, 2006; Unützer *et al.* 2006; Montgomery *et al.* 2010; Fuller *et al.* 2011), ENACT-type tools can be used to objectively evaluate performance for certification or employment. Objective demonstration of achieving and maintaining competency may be helpful to assure that these cadres of workers can be appropriately compensated for providing services.

### Limitations

The current results are limited to formative work done in Nepal. Additional sites and types of programs will need to adapt competency tiers based on their setting and implementation context. Although a three-tiered response scale appeared to have feasibility and utility from a qualitative perspective in Nepal, quantitative psychometric studies are needed to evaluate distribution of scores and pre-/post-training changes. Of note, initial qualitative process evaluation of the ENACT tool in Liberia suggested that a four-tiered response scale may be needed to prevent ceiling effects and enable non-specialists to track improvement over longer periods of time while providing mental health services (that is, to have a higher level to strive for as opposed to communicating a message that a third-tier score is terminally sufficient). Another component to incentive further learning through peer non-specialist ratings is the use of treatment-specific scales appropriate for non-specialists that address the five challenges raised here.

Important next steps will include evaluating the ability to differentiate among the tiers when rating the transcripts in Nepali rather than English, and when rating direct observation or video files. Lack of blinding to pre- and post-training status is a major limitation for the quantitative analysis of the scores. The lack of blinding likely biases raters to inflate scores after training, and potentially depress scores prior to training because of unconscious biases regarding what participants should be able to do at different stages. Similarly, there may be social desirability bias for inflated post-training scores because raters want scores to reflect positively on the trainers. Therefore, blinding is currently being used for coding comparisons between pre- *v.* post-training competence. Similarly, for other training and supervision studies, objective evaluations of competence should include blinded ENACT ratings in addition to those completed during trainings. Ultimately, the utility of the scale and the proposed three-tier rating categories needs to be assessed against client outcomes. This work is currently underway through PRIME in Nepal. The process documented here represents the crucial preliminary steps toward analyzing the pathway from training to client well-being in task-sharing initiatives.

## Conclusion

With increasing attention to task-sharing in global mental health, there is a need to assure that non-specialists achieve and maintain a minimum standard of competence and quality in order to adhere to evidence-based practices, strive for positive client outcomes, and reduce risk of harm. Lack of human resources in the form of mental health experts is a challenge not only for service delivery but also for training and supervision. Task-sharing aspect of training and supervision so that non-specialists can improve quality among their peers is one strategy to improve implementation and dissemination. Development of tools for non-specialists to use with peers can facilitate this process. We identified five challenges for development and use of non-specialist peer rating tools and proposed approaches to address these challenges as demonstrated through development of the ENACT scale. Ultimately, these challenges and the need for tools such as ENACT are not limited to low-resource setting. These are needs in high-resource settings where task-sharing is occurring as well. Future work is required for development and implementation of user-friendly, value added peer-rating system across settings varied by culture, income, and mental health needs.

## Supplementary material

For supplementary material accompanying this paper visit http://dx.doi.org/10.1017/gmh.2015.21. click here to view supplementary material
