# Supplementary material for: Development of a scoring system for non-specialist ratings of clinical competence in global mental health: a qualitative process evaluation of the Enhancing Assessment of Common Therapeutic Factors (ENACT) scale
Source: Glob Ment Health (Camb). 2015 Dec 9;2:e23. doi: 10.1017/gmh.2015.21 (PMC5269630; doi:10.1017/gmh.2015.21)
Supplement: Supplementary file 1 [file S2054425115000217sup001.docx]

**ENhancing Assessment of Common Therapeutic factors (ENACT)**

| **ITEM 1. NON-VERBAL COMMUNICATION & ACTIVE LISTENING: EYE CONTACT, FACIAL EXPRESSION, BODY LANGUAGE & GESTURES** |
| --- |
| ⃝ 1 NEEDS IMPROVEMENT = does not make any eye contact or stares at patient; shows anger; laughs at patient; mocks patient; turns away from patient; repeatedly interrupts patient; ignores patient; answers mobile phone without permission |
| ⃝ 2 DONE PARTIALLY = does not consistently use body language to express interest; rarely makes eye contact; shows limited emotion; appears artificial |
| ⃝ 3 DONE WELL = makes appropriate eye contact throughout interaction; smiles when appropriate; sits at appropriate angle from patient and leans in to show interest; use of ‘uh-huh’, ‘hmm’ or other culturally appropriate non-lexical utterances to signal interest |
| **ITEM 2. VERBAL COMMUNICATION SKILLS: OPEN-ENDED QUESTIONS, SUMMARIZING & CLARIFYING STATEMENTS** |
| ⃝ 1 NEEDS IMPROVEMENT = uses mostly ‘yes/no’ questions, e.g., “Will you? Can you?” |
| ⃝ 2 DONE PARTIALLY = uses open-ended questions but does not explore topics further or offer summaries for patient reflection |
| ⃝ 3 DONE WELL = uses open-ended questions, summarizing and clarifies statements, e.g., “What happened? Tell me more.” |
| **ITEM 3. RAPPORT BUILDING & SELF-DISCLOSURE** |
| ⃝ 1 NEEDS IMPROVEMENT = clinician does not introduce him/herself or attempt to make the patient feel comfortable or clinician dominates the session talking about his/her own experiences |
| ⃝ 2 DONE PARTIALLY = clinician introduces him/herself but does not help the patient feel comfortable through small talk and informal conversation or clinician disclosure but it is not related to patient experience or needs |
| ⃝ 3 DONE WELL = clinician introduces him/herself, tries to make the patient feel comfortable and disclosure focuses on patient needs |
| **ITEM 4. EXPLORATION, INTERPRETATION & NORMALIZATION OF FEELINGS** |
| ⃝ 1 NEEDS IMPROVEMENT = clinician does not ask about patient’s feelings or clinician is judgmental/critical about patient’s emotions and feelings (e.g., “You shouldn’t feel that way”, “You should stop thinking or feeling that.”) |
| ⃝ 2 DONE PARTIALLY = clinician asks about feelings but does not normalize/validate or does not explore feelings in detail with patient |
| ⃝ 3 DONE WELL = clinician explains that the patient’s feelings in context and if appropriate, feelings are expected for a person in his/her situation |
| **ITEM 5. DEMONSTRATION OF EMPATHY, WARMTH & GENUINENESS** |
| ⃝ 1 NEEDS IMPROVEMENT = clinician is critical, hostile, or dismissive of patient’s concerns or complaints |
| ⃝ 2 DONE PARTIALLY = clinician is generally warm and friendly to patient, but does not demonstrate the ability to put him/herself in the experience of the patient |
| ⃝ 3 DONE WELL = clinician demonstrates that he/she understands the experience of patient in a genuine and sincere manner |
| **ITEM 6. ASSESSMENT OF FUNCTIONING & IMPACT ON LIFE** |
| ⃝ 1 NEEDS IMPROVEMENT = clinician does not ask the patient about the impact on functioning and daily life from feelings, thoughts, psychosocial problems, etc. |
| ⃝ 2 DONE PARTIALLY = clinician asks about functioning and daily life activities but does not connect it to psychosocial/mental health concerns |
| ⃝ 3 DONE WELL = clinician explores the relationship between psychosocial problem and functioning |
| **ITEM 7. EXPLORATION OF PATIENT'S & SOCIAL SUPPORT NETWORK'S EXPLANATION FOR PROBLEM (CASUAL & EXPLANATORY MODELS)** |
| ⃝ 1 NEEDS IMPROVEMENT = clinician does not ask the patient about his/her own view of the cause or is judgmental/critical about patient’s explanation (e.g. “Witchcraft doesn’t cause these problems, that is an ignorant/backwards idea!) |
| ⃝ 2 DONE PARTIALLY = clinician asks patient about his/her own view of cause but does not explore if this the same as the family’s view |
| ⃝ 3 DONE WELL = clinician asks the patient about cause and asks if family/support network have similar or different explanations |
| **ITEM 8. INCORPORATION OF COPING MECHANISMS & PRIOR SOLUTIONS** |
| ⃝ 1 NEEDS IMPROVEMENT = clinician does not ask the patient about how he/she has coped or clinician is judgmental about how patient has coped (e.g., “Why did you think that work?” or “That isn’t helpful.”) |
| ⃝ 2 DONE PARTIALLY = clinician asks about coping and prior solutions, but does not provide positive feedback |
| ⃝ 3 DONE WELL = clinician asks about coping and provides positive feedback in regard to agency or pathways thinking |
| **ITEM 9. ASSESSMENT OF PATIENT'S RECENT LIFE EVENTS & ACKNOWLEDGEMENT OF IMPACT ON PSYCHOSOCIAL WELLBEING** |
| ⃝ 1 NEEDS IMPROVEMENT = clinician does not ask about triggering life events |
| ⃝ 2 DONE PARTIALLY = clinician asks about life events but does not connect with current mental health issues |
| ⃝ 3 DONE WELL = clinician asks about life events and discusses connection with current mental health |
| **ITEM 10. ASSESSMENT OF OTHER MENTAL HEALTH PROBLEMS, ALCOHOL/DRUG USE & PHYSICAL HEALTH PROBLEMS** |
| ⃝ 1 NEEDS IMPROVEMENT = clinician does not ask about any related conditions, e.g., alcohol or drug use, physical health problems, injuries, head trauma, medications, etc. |
| ⃝ 2 DONE PARTIALLY = clinician takes partial history but does not explore positive responses in relation to mental health, e.g., clinician does not connect other health problems or substance use to current mental health |
| ⃝ 3 DONE WELL = clinician assesses related health issues and explains relationship to patient’s condition when appropriate |
| **ITEM 11. APPROPRIATE INVOLVEMENT OF FAMILY MEMBERS & OTHER CAREGIVERS** |
| ⃝ 1 NEEDS IMPROVEMENT = *When family member is present*: clinician ignores family during session or clinician only talks to family members and ignores patient; *When family member is not present*: clinician does not ask about family at all |
| ⃝ 2 DONE PARTIALLY = *When family member is present*: clinician interviews both patient and family but does not facilitate interaction between family and patient during session; *When family member is not present*: clinician asks about family involvement but does not explore patient’s reasons or preferences for involvement or non-involvement |
| ⃝ 3 DONE WELL = *When family member is present*: clinician helps both patient and family participate and encourages interaction between them; *When family member is not present*: clinician explores preferred family engagement with the patient and does role-plays or coaching |
| **ITEM 12. COLLABORATIVE GOAL SETTING & ADDRESSING PATIENT’S EXPECTATIONS** |
| ⃝ 1 NEEDS IMPROVEMENT = clinician does not ask patient about his/her goals and expectations for treatment, or clinician just tells patient what to do without asking his/her expectations |
| ⃝ 2 DONE PARTIALLY = clinician asks patient about goals but does not discuss if these are realistic or can be accomplished |
| ⃝ 3 DONE WELL = clinician asks about goals and discusses with patient what is and is not achievable through treatment, and clinician and patient collaboratively establish treatment plan |
| **ITEM 13. PROMOTION OF REALISTIC HOPE FOR CHANGE** |
| ⃝ 1 NEEDS IMPROVEMENT = clinician either gives no hope (e.g., you will never get better) or gives unrealistic expectations (e.g., you will be cured in a few weeks and never have problems again) for what to expect in treatment and recovery |
| ⃝ 2 DONE PARTIALLY = clinician vaguely tells patient what will happen during treatment |
| ⃝ 3 DONE WELL = clinician helps patient feel positive about the future and creates realistic expectations about what can and cannot be achieved through treatment, and clinician checks patient’s understanding of realistic change |
| **ITEM 14. PSYCHOEDUCATION INCORPORATING LOCAL (ETHNOPSYCHOLOGICAL) CONCEPTS & TERMS** |
| ⃝ 1 NEEDS IMPROVEMENT = clinician uses technical jargon to explain mental health or uses stigmatizing terms or does not explain how treatment works |
| ⃝ 2 DONE PARTIALLY = clinician uses a limited amount of technical jargon and no stigmatizing terms, but clinician does not incorporate patient’s explanatory model or other local psychological concepts into psychoeducation |
| ⃝ 3 DONE WELL = clinician conducts psychoeducation using local psychological concepts including patient’s explanatory model (see Item 7), local terminology, and idioms of distress to explain mental health and treatment in non-stigmatizing language, and checks to see if patient understands |
| **ITEM 15. USE OF PROBLEM SOLVING STEPS: PROBLEM FORMULATION, PRIORITIZATION, SOLUTION GENERATION & ACTION PLANNING** |
| ⃝ 1 NEEDS IMPROVEMENT = clinician does work with patient to formulate key problem requiring help, support, or treatment |
| ⃝ 2 DONE PARTIALLY = clinician helps patient formulate and prioritize key problem, but does not complete steps #2-4 (see below) |
| ⃝ 3 DONE WELL =clinician helps patient (1) formulate and prioritize primary problem, (2) brainstorm solutions, (3) explores advantages and disadvantages, and (4) formulate action plan |
| **ITEM 16. ELICITATION OF FEEDBACK WHEN PROVIDING ADVICE, SUGGESTIONS & RECOMMENDATIONS** |
| ⃝ 1 NEEDS IMPROVEMENT = clinician lectures patient about what to do without asking if this is acceptable and comfortable for the patient, or clinician does not give any suggestions at all |
| ⃝ 2 DONE PARTIALLY = clinician gives focused advice but does not ask for feedback from patient to see if the advice is helpful |
| ⃝ 3 DONE WELL = clinician gives a few suggestions when asked by patient and asks for patient feedback about suggestions |
| **ITEM 17. EXPLAINATION AND PROMOTION OF CONFIDENTIALITY** |
| ⃝ 1 NEEDS IMPROVEMENT = clinician does not address confidentiality or does not adjust topics of discussion based on setting |
| ⃝ 2 DONE PARTIALLY = clinician tells patient that everything is confidential without explaining exceptions such as harm to self or others, or clinician states everything is confidential while conducting session in non-private setting |
| ⃝ 3 DONE WELL = clinician explains that all clinician-patient discussions are confidential with the exception of harm to self and others, and clinician adjusts conversation topics based on private or non-private setting |
| **ITEM 18. ASSESSMENT OF HARM TO SELF, HARM TO OTHERS, HARM FROM OTHERS & DEVELOPING COLLABORATIVE RESPONSE PLAN** |
| ⃝ 1 NEEDS IMPROVEMENT = clinician does not ask about harm to self or others |
| ⃝ 2 DONE PARTIALLY = clinician asks about harm to self or others, but does not help patient develop a plan for safety |
| ⃝ 3 DONE WELL = clinician asks about harm to self or others and facilitates appropriate planning and actions to assure safety |

This tool is a basic framework and is intended for translation, modification, and refinement based on needs for specific trainings and interventions and the cultural context. Please contact Brandon Kohrt, MD, PhD, Duke Global Health Institute [brandon.kohrt@duke.edu](mailto:brandon.kohrt@duke.edu) for additional training and coding materials and for assistance in transcultural translation and adaption procedures.

*ENACT citation*: Kohrt, B. A., Jordans, M. J. D., Rai, S., Shrestha, P., Luitel, N. P., Ramaiya, M., Singla, D. Patel, V. (2015). Therapist Competence in Global Mental Health: Development of the Enhancing Assessment of Common Therapeutic Factors (ENACT) Rating Scale. *Behaviour Research and Therapy, 69*, 11-21. doi: <http://dx.doi.org/10.1016/j.brat.2015.03.009>
